# Traumatic brain injury in Brazil: an epidemiological study and systematic review of the literature

**DOI:** 10.1590/0004-282X-ANP-2021-0035

**Published:** 2022-04-20

**Authors:** Ana Luísa Gonçalves MAGALHÃES, João Luís Vieira Monteiro de BARROS, Maíra Glória de Freitas CARDOSO, Natália Pessoa ROCHA, Rodrigo Moreira FALEIRO, Leonardo Cruz de SOUZA, Aline Silva de MIRANDA, Antônio Lúcio TEIXEIRA

**Affiliations:** 1 Universidade Federal de Minas Gerais, Faculdade de Medicina, Laboratório Interdisciplinar de Investigação Médica, Belo Horizonte MG, Brazil.; 2 University of Texas Health Science Center at Houston, McGovern Medical School, Texas, USA.; 3 Fundação Hospitalar do Estado de Minas Gerais, Hospital João XXIII, Belo Horizonte MG, Brazil.; 4 Universidade Federal de Minas Gerais, Instituto de Ciências Biológicas, Departamento de Morfologia, Laboratório de Neurobiologia, Belo Horizonte MG, Brazil.; 5 Santa Casa Belo Horizonte, Ensino e Pesquisa, Belo Horizonte MG, Brazil.

**Keywords:** Brain Injuries, Traumatic, Brain Concussion, Epidemiology, Brazil, Lesões Encefálicas Traumáticas, Concussão Encefálica, Epidemiologia, Brasil

## Abstract

Background: Traumatic brain injury (TBI) is a serious public health problem worldwide. Although TBI is common in developing countries, there are few epidemiological studies. Objective: To investigate the sociodemographic and clinical features of patients with TBI at the Hospital João XXIII, a public reference center for trauma in Belo Horizonte, Brazil, and to systematically review the available literature on TBI in Brazil. Methods: Clinical and sociodemographic data were collected from electronic medical records for the entire month of July 2016. The literature on epidemiology of TBI in Brazil was systematically reviewed using MeSH/DeCS descriptors in the PubMed and Lilacs databases. Results: Most patients admitted with TBI were male and under 60 years of age. Mild TBI was the most prevalent form and the most common cause of TBI was falls. A Glasgow Coma Scale score below 12, neuroimaging changes on computer tomography, and presence of any medical conditions were significantly associated with longer hospital stay. Brazilian studies showed that TBI affected mainly men and young adults. In addition, mild TBI was the most common TBI severity reported and the most common causes were motor vehicle accidents and falls. Conclusions: Overall, the profile of TBI in this center reflects the data from other Brazilian studies.

## RESUMO

Antecedentes: O traumatismo cranioencefálico (TCE) representa, mundialmente, um problema sério de saúde pública. Apesar de o TCE ser prevalente em países em desenvolvimento, estudos epidemiológicos permanecem escassos. Objetivo: Investigar as características sociodemográficas e clínicas de pacientes acometidos por TCE no Hospital João XXIII - centro de referência em trauma situado em Belo Horizonte, Brasil - e revisar sistematicamente toda a literatura disponível sobre o TCE no Brasil. Métodos: Os dados clínicos e sociodemográficos foram coletados apenas para o mês de julho, 2016, por meio de prontuários eletrônicos. A literatura sobre a epidemiologia do TCE no Brasil foi sistematicamente revisada usando descritores *Medical Subject Headings* (MeSH)/Descritores em Ciências da Saúde (DeCS) nos bancos de dados PubMed e Literatura Latino-Americana e do Caribe em Ciências da Saúde (Lilacs). Resultados: Os pacientes acometidos por TCE eram em sua maioria homens com menos de 60 anos. O TCE leve foi a gravidade mais prevalente entre os casos. O TCE foi causado principalmente por quedas. Escores menores que 12 na escala de Coma de Glasgow mais alterações de neuroimagem em tomografia computadorizada e a presença de qualquer comorbidade médica estão significativamente associados à maior estadia hospitalar. Estudos brasileiros demonstraram que o TCE acomete principalmente homens e adultos jovens. Além disso, o TCE leve foi a gravidade mais comum reportada, e os mecanismos de TCE mais comuns foram acidentes automobilísticos e quedas. Conclusões: O perfil de pacientes acometidos por TCE no centro de referência em questão reflete os dados de outros estudos brasileiros.

## INTRODUCTION

Traumatic brain injury (TBI) is defined as an injury caused by external force to the head that results in an anatomical lesion or functional impairment of cranial or encephalic structures. TBI is the leading cause of morbidity and mortality in polytrauma patients and is one of the main causes of death in individuals under 45 years of age^1^ ^,^ ^2^ ^,^ ^3^. TBI can have a variety of causes, from falls to car accidents.

Because of its medical and socioeconomic burden, TBI is a major public health problem worldwide. In the United States, 2.8 million emergency department visits were due to TBI and approximately 124,000 of the most severe cases develop long-term impairment^4^ ^,^ ^5^. In a single North American state, the annual direct medical cost of TBI was estimated at $95 million, or $1.67 million per 100,000 people^6^. Although lifetime costs for patients with TBI vary according to their demographic characteristics, the costs in Canadian dollars (CAD) for nonfatal cases was estimated at $2,318 for males and $2,200 for females^6^. In Europe, TBI accounted for 37% of all injury-related deaths and was estimated to cost a total of €22,907 million in 2010^7^ ^,^ ^8^. Limited demographic and socioeconomic information on TBI is available from developing countries^9^.

Although TBI is widespread in Brazil and seems to have an economic and social impact, there are very few epidemiological studies ^10^ ^,^ ^11^. A previous study reported that 40% of deaths in patients aged 5 to 9 years in Brazil are due to TBI and that for every patient who dies, there are at least another three more patients with long-term sequelae^12^. In addition, the annual cost of hospitalizations due to TBI has been estimated at approximately R$ 156,300,000 (US$ 70,960,000)^11^. Unfortunately, these estimates may not reflect the actual Brazilian reality, due in part to a high rate of unreported cases associated with immediate death and the absence of a nearby emergency unit^11^ ^,^ ^13^ ^,^ ^14^. Furthermore, reliable quantification of the impact caused by TBI is usually not accurate because measurements are not standardized and data collection on the incidence and outcome of brain injury is incomplete. Therefore, clinical-epidemiological studies are urgently needed to systematically investigate TBI in Brazil.

The current study aimed to investigate sociodemographic and clinical characteristics of patients admitted to João XXIII Hospital with TBI and to identify factors that may influence TBI morbidity and mortality. Also, the epidemiological data available on TBI in Brazil was systematically review.

## METHODS

### Original report

This was an observational study conducted at the Joao XXIII Hospital. This hospital is the main trauma center in the Metropolitan region of Belo Horizonte, the third largest metropolitan region in Brazil with more than five million inhabitants. The study was approved by the Human Research Ethics Committee of the Federal University of Minas Gerais (COEP-UFMG).

All records of patients admitted to the Emergency Department of the Joao XXIII Hospital within one month (July 2016) were evaluated using a structured protocol to obtain sociodemographic and clinical information. The sociodemographic data included: (i) sex, (ii) ethnicity, (iii) marital status, (iv) place of residence (Belo Horizonte, metropolitan area, rural area), and (v) educational level. Clinical variables included TBI features (Glasgow Coma Scale Score [GCS], CT neuroimaging changes, hemodynamic instability, and ventilatory support) and hospital outcome. The following premorbid variables were also recorded: (i) clinical comorbidities (any medical conditions that were either secondary to the TBI or that the patient already had on admission) and (ii) alcohol or illicit drug use (assessed via medical record). The causes, severity, and type of TBI were also recorded. Neuroimaging results were included when available.

Exclusion criteria included: (i) follow-up patients, (ii) non-TBI patients (evaluated via the absence of a TBI diagnosis on record), (iii) burn victims, (iv) exogenous intoxications, (v) venomous animal bites, (vi) trauma patients without TBI, and (vii) patients admitted 24 hours after TBI.

Statistical analyses were conducted with *Statistical Package for the Social Sciences* (SPSS) software, version 17.0. Chi-squared analyses were performed to determine statistically significant frequencies of specific events in subgroups. Binary logistic regression using a backward elimination approach was performed to determine which variables were significantly associated with a longer hospital stay, defined as more than 24 hours, as opposed to patients discharged within 24 hours after hospital admission. At the João XXIII Hospital, patients whose state of consciousness remained stable for 24 hours were discharged. The following variables were included in the initial model: age, sex, GCS score (greater than or equal to 13 or less than 12), comorbidity (presence or absence), neuroimaging changes in computed tomography, and alcohol and drug use. Stepwise backward selection was performed automatically using the SPSS software, version 17.0 (SPSS Inc., Chicago, IL, USA), and exclusion testing was done with the likelihood ratio based on the conditional parameter estimates. The goodness of fit of the logistic regression model was assessed using the Hosmer-Lemeshow test and a Receiver Operating Characteristic (ROC) curve.

### Systematic review

A systematic search for TBI studies in Brazil was performed independently by two authors (JLVMB and ASM) in the PubMED and Lilacs databases using the MeSH/DeCS descriptors for *traumatic brain injury, *epidemiology, and *Brazil. The inclusion criteria were as follows: (i) studies evaluating sociodemographic and clinical information on TBI cases in Brazil, (ii) original articles, and (iii) articles in Portuguese, Spanish, or English.

## RESULTS

In July 2016, 6,184 patients were admitted to the hospital, with 490 individuals diagnosed with TBI. These 490 individuals accounted for 7.92% of the total admissions during the research period. Four hundred seventy-seven records had enough information to determine clinical outcome by age, while 436 records contained all information required by our research protocol (data not shown).

Male patients formed the majority of our sample (n=324, 66.1%). Most TBI occurred in adults (n=259, 52.9%). The most common mechanism for TBI was an unspecified fall (n=124, 25.3%), followed by a fall from one’s own height (n=118, 24.1%) (Table 1).

**Table 1.** Sociodemographic data of the 490 available traumatic brain injury records.

		n	%
Sex	Male	324	66.1
	Female	166	33.9
Origin	Belo Horizonte	341	69.9
	Metropolitan region	107	21.8
	Metropolitan region outskirts	4	0.8
	Outside metropolitan region (but still within the state of Minas Gerais)	29	5.9
	Different State	2	0.4
	Not informed	7	1.4
Age	0-18 years	149	30.4
	19-59 years	259	52.9
	60 years or more	82	16.7
Race	Brown	336	68.6
	White	114	23.3
	Black	32	6.5
	Not informed	8	1.6
Outcome	Death	15	3.1
	Discharge<24h	367	74.9
	Discharge >24h	95	19.4
	Hospitalized	7	1.4
	Not informed	6	1.2
TBI mechanism	Unspecified fall	124	25.3
	Fall from own height	118	24.1
	Fall from superior height	43	8.8
	Aggression	61	12.4
	Firearm	6	1.2
	Hit or struck by a car	45	9.2
	Traffic collision	66	13.5
	Non-traffic-related collision	16	3.3
	Repetitive TBI	4	0.8
	Not informed	7	1.4

TBI: traumatic brain injury.

The consequences of TBI differed considerably between age ranges (p=0.031). Deaths by age range were: (i) ≤18 years old, 1 death/112 individuals (0.89%), (ii) 19-59 years old, 7 deaths/245 individuals (2.9%), and (iii) ≥60 years old, 6 deaths/79 individuals, (7.6%). For the latter analysis, we considered only the 436 records that contained all the data required by our research protocol.

Patients with TBI were divided into three groups based on their GCS score on hospital admission. Patients who had GSC scores of 13-15 on hospital admission were classified as “mild TBI”. Patients with GSC scores of 9-12 and 3-8 were classified as “moderate TBI” and “severe TBI”, respectively^11^ ^,^ ^13^. Patients with mild TBI accounted for the majority of TBI-related admissions and comprised 87.4% of the total number of TBI cases. Moderate and severe TBI cases accounted for 5.5 and 7.1% of TBI cases, respectively.

Next, we analyzed the mechanisms involved in TBI. The mechanisms of TBI were differed significantly between the different severity categories of TBI. Unspecified fall and traffic accident were the most frequent mechanisms for mild and severe TBI, respectively (data not shown).

Male patients were the most affected by TBI across severity levels (p=0.022). We also analyzed the incidence of comorbidities, CT neuroimaging changes, hemodynamic instability, ventilatory support, and death across TBI severity levels (Table 2). Severe TBI accounted for the majority of deaths (57.1%), whereas mild and moderate TBI accounted for 21.4% each. These deaths were related to TBI or TBI-associated injuries.

**Table 2.** Clinical variables across different traumatic brain injury severities.

		GCS Score			p-value
		Mild (13 to 15)	Moderate (12 to 9)	Severe (8 to 3)	
Sex	Male	240 (63%)	19 (20.1%)	5 (16.1%)	0.022
	Female	141 (37%)	5 (79.2%)	26 (83.9%)	
Use of drugs		12 (3.1%)	2 (8.7%)	1 (3.2%)	0.36
Alcohol		71 (18.6%)	9 (37.5%)	3 (9.7%)	0.029
Comorbidity		95 (25%)	6 (25%)	2 (6.5%)	0.065
CT neuroimaging findings		39 (11.7%)	13 (54.2%)	24 (77.4%)	<0.001
Hemodynamic instability		0 (0%)	2 (8.3%)	4 (13.8%)	<0.001
Ventilation Support		4 (1.1%)	6 (25%)	24 (80%)	<0.001
Outcome	Death	3 (0.8%)	3 (13.6%)	8 (29.6%)	<0.001
	Discharge>24h	61 (16.3%)	12 (54.5%)	16 (59.3%)	
	Discharge<24h	311 (82.9%)	7 (31.8%)	3(11.1%)	

GCS: Glasgow coma scale; TBI: traumatic brain injury; CT: computed tomography.

**Figure 1.**
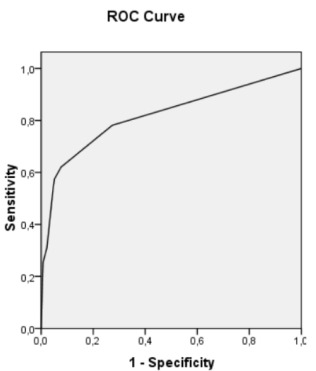
ROC curve of the logistic regression model (AUC=0.819).

In multivariate analysis, CT neuroimaging changes, the presence of medical comorbidities, and a GCS score of 12 or less remained as significant factors associated with longer hospital stay (>24h). The results are presented in Table 3. The logistic regression model was significant [Hosmer-Lemeshow goodness of fit test (step 5): chi-square=3.177; p=0.204] and predicted variability yielded an area under the curve (AUC) of 0.819 in the ROC analysis (Figure 1).

**Table 3.** Logistic model analysis to predict hospital admission for more than 24 hours.

Predictive variable	B	SE	Wald	df	p-value	OR	95%CI for OR	
							Lower	Upper
CT neuroimaging changes	-2.909	0.378	59.220	1	0.000	0.055	0.026	0.114
Medical comorbidity	-0.703	0.347	4.115	1	0.043	0.495	0.251	0.977
GCS score	-1.838	0.491	13.998	1	0.000	0.159	0.061	0.417

CT: computed tomography; GCS: Glasgow coma scale; B: beta coefficient; SE: standard error; df: degrees of freedom; OR: *Odds Ratio*; 95%CI: 95% confidence interval.

**Figure 2.**
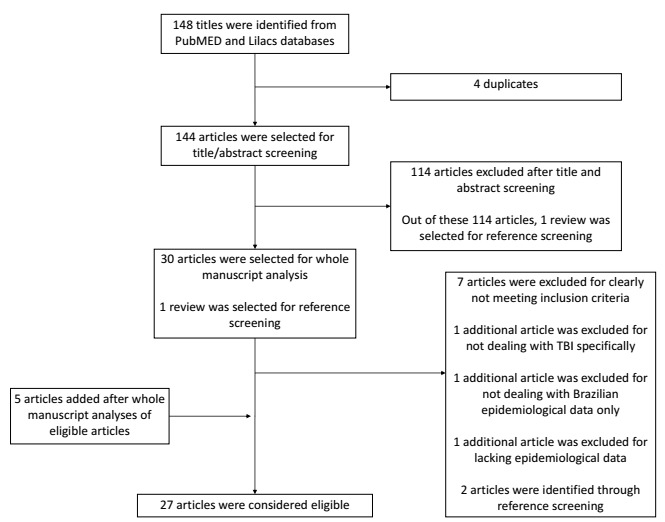
Flowchart of study selection process.

In our systematic review, we first identified 148 possible titles in the PubMED and Lilacs databases. Four articles were duplicates, and 114 studies were excluded after title/abstract screening. Of these 114 articles, we set aside one review for further reference screening. Thirty articles were fully analyzed, and 10 of these either did not meet our inclusion criteria or did not contain the required information. Two additional articles were identified in the references of review studies. Also, five additional articles were identified while reading the selected manuscripts, giving us a total of 27 eligible articles (Figure 2).

TBI: traumatic brain injury.

Most studies were conducted in cities in the state of São Paulo (n=6)^15^ ^,^ ^16^ ^,^ ^17^ ^,^ ^18^ ^,^ ^19^ ^,^ ^20^. Three studies dealt exclusively with epidemiological data on patients who developed specific sequelae as a result of TBI, including diffuse axonal injury, intracranial hypertension, and hypoxic brain damage^15^ ^,^ ^21^ ^,^ ^22^. Two studies addressed epidemiological data on patients affected by specific TBI mechanisms, such as falls from their own height and firearm bullets^19^ ^,^ ^23^. In most studies, mild TBI was found to be the most prevalent type (n=10)^16^ ^,^ ^17^ ^,^ ^23^ ^-^ ^30^. Additionally, young men were most commonly affected in all studies^11^ ^,^ ^15^ ^-^ ^40^. There was limited information on ethnicity, with only three studies providing this information^11^ ^,^ ^15^ ^,^ ^35^. Traffic/vehicle accidents were the most common mechanism for TBI, followed by falls^15^ ^-^ ^17^ ^,^ ^19^ ^,^ ^21^ ^,^ ^24^ ^-^ ^31^ ^,^ ^33^ ^-^ ^40^. This information is presented in Table 4.

**Table 4.** Sociodemographic characteristics of Brazilian epidemiological studies on traumatic brain injury.

Reference	Location	Study design	TBI severity/type	Most Common TBI severity (if applicable)	Male		Female		Most afflicted age group	Most afflicted ethnicity	Death	Most common TBI mechanism
					n	%	n	%				
Melo et al., 2019^24^	Parnaíba, Piauí, Brazil	Retrospective and Descriptive	General	Mild, n=50 (42.7%)	94	80.3%	23	19.7%	Mean age: 33.17 years (SD±17.2)	Not informed	Not informed	Automobile accidents, n=96 (82.1%)
Marinho et al., 2017^31^	Natal, Rio Grande do Norte, Brazil	Cross-sectional	General	Moderate, n=228 (61.3%)	317	85.2%	55	14.8%	18-30 years old, n=209 (56.2%)	Not informed	Not informed	Automobile accidents, n=95 (25.6%)
De Almeida et al., 2016^11^	Not applicable	Cross-sectional	General	Not informed	97,552 (mean per year)	77.7% (mean per year)	28,017 (mean per year)	22.3 (mean per year)	20-29 years old, n=28,905.4 (mean per year)	Mixed race, n=3,142,782.4* (mean per year) *Greater number of cases because data were missing	n=9,714 (7.7%)(mean per year)	Not informed
Vieira et al., 2016^15^	São Paulo, São Paulo, Brazil	Prospective Cohort Study	Severe TBI with diffuse axonal injury	Not applicable	70	89.7%	8	10.3%	18-28 years old, n=34 (43.6%)	White, n=51 (65.4%)	n=24 (30.8%)	Traffic accidents, n=65 (83.3%)
Tavares et al., 2014^32^	Distrito Federal, Brasília, Brazil	Cross-sectional	General	Severe, n=108 (55.7%)	161	82.99%	33	17.01%	21-40 years old, n=67 (34.5%)	Not informed	Not informed	Physical aggression, n=57 (29.4%)
De Souza et al., 2013^19^	São Paulo, São Paulo, Brazil	Cross-sectional	General TBI caused by firearm projectiles	Severe, n=68 (37.6%)	154	85%	27	15%	21-30 years old, (47%)	Not informed	Not informed	Not applicable
Santos et al., 2013^25^	Pelotas, Rio Grande do Sul, Brazil	Epidemiological, Descriptive, and Retrospective	General	Mild, n=202 (40.7%)	314	63.3%	182	36.7%	0-15 years old, n=220 (44.3%)	Not informed	n=2 (0.4%)	Falls, n=233 (47.0%)
Fernandes et al., 2013^33^	Not applicable	Cross-sectional, descriptive	General	Not informed	358,780	81.5%	81,706	18.5%	14-34 years old, n=231,827 (53.0%)	Not informed	n=52,087 (12.0%)	Falls, n=154,170 (35.0%)
Carvalho Viégas et al., 2013^34^	Ananindeua, Pará, Brazil	Epidemiological, Cross-sectional, observational	General	Not informed	220	88%	30	12%	20-30 years old, n=81 (32.4%)	Not informed	n=55 (22%)	Traffic accidents, n=91 (36.4%)
Ruy and Rosa, 2011^35^	Criciúma, Santa Catarina, Brazil	Cross-sectional, descriptive, retrospective	General	Severe, n=63 (67.7%)	82	88.2%	11	11.8%	Mean age: 34.6 years (SD±16.7)	White n=84 (90.3%)	n=25 (26.9%)	Automobile accidents, n=52 (55.9%)
Moura et al., 2011^26^	Petrolina, Pernambuco, Brazil	Cross-sectional, epidemiological	General	Mild, n=54 (53.47%)	87	86.14%	14	13.86%	21-40 years old, n=52 (51.49%)	Not informed	n=8 (7.92%)	Motorcycle accident, n=45 (44.55%)
Ramos et al., 2010^36^	Caruaru, Pernambuco, Brazil	Document-based	General	Not informed	139	81.2%	32	18.7%	25-49 years old, n=56 (29.9%)	Not informed	Not informed	Motorcycle accident, n=34 (19.9%)
Guerra et al., 2010^21^	Belo Horizonte, Minas Gerais, Brazil	Retrospective cohort study	General TBI patients who developed intracranial hypertension	Severe, n=132 (100%)	89	67.4%	43	32.6%	7-9 years old	Not informed	n=68 (51.5%)	Getting hit by a vehicle, n=68 (51.5%)
Martins et al., 2009^37^	Florianópolis, Santa Catarina, Brazil	Prospective	Severe	Not applicable	631	84%	117	15.6%	Mean age: 34.8 years old (SD±16.3)	Not informed	n=249 (33.3%)	Road accident, n=225 (30.1%)
Braga et al., 2008^23^	Florianópolis, Santa Catarina, Brazil	Prospective	General TBI caused by one’s own height	Mild, n=69 (90.7%)	44	57.9%	32	42.1%	Mean age for men: 44.7 years Mean age for women: 47.2	Not informed	Not informed	Not applicable
Faria et al., 2008^38^	Uberlândia, Minas Gerais, Brazil	Epidemiological, Prospective	General (Severe and moderate were grouped together)	Severe and moderate (grouped together), n=56 (66.7%)	68	80.9%	16	19.1%	Mean age for severe and moderate: 40.6 years Mean age for mild: 34.8	Not informed	Not informed	Transport accidents, n=54 (64.74%)
Pereira et al., 2006^27^	Aracaju, Sergipe, Brazil	Longitudinal Prospective	General	Mild, n=422 (89%)	344	73%	126	27%	10-29 years old	Not informed	n=3 (0.6%)	Accidental fall, n=148 (31.5%)
Melo et al., 2006^28^	Salvador, Bahia, Brazil	Cross-sectional descriptive	General	Mild, n=249 (63.8%)	280	71.8%	110	28.2%	Not applicable (Study conducted on a specific group age (0-19 years old)	Not informed	Not informed	Fall from height, n=134 (34.4%)
Melo et al., 2004^29^	Salvador, Bahia, Brazil	Cross-sectional	General	Mild, n=146 (38.4%)	460	82.9%	95	17.1%	21-30 years old, n=128 (23.2%)	Not informed	n=127 (22.9%)	Traffic accidents, n=226 (40.7%)
Dantas Filho et al., 2004^39^	Campinas, São Paulo, Brazil	Cross-sectional	Severe	Not applicable	166	80.58%	40	19.42%	Mean age: 29.21 years old	Not informed	n=75 (36.40%)	Traffic accidents, n=147 (71.36%)
Gusmão et al., 2002^22^	Belo Horizonte, Minas Gerais, Brazil	Prospective	Fatal TBI victims	Not applicable	90	75.0%	30	25.0%	Mean age: 37.5 years old (SD±18.3)	Not informed	Not applicable (Post-mortem study)	Not applicable (all patients came from traffic accidents)
Koizumi et al., 2001^40^	Not applicable	Cross-sectional	General	Not informed	10,251	62.6%	6,125	37.4%	(Study conducted on children who were ≥ 10 years old) 0-4 years old, n=9,302 (56.8%)	Not informed	n=332 (2.0%)	Falls, n=10,022 (61.2%)
Koizumi et al., 2000^20^	São Paulo, São Paulo, Brazil	Cross-sectional, retrospective	General	Not informed	2784	76.6%	851	23.41%	≤10 years old (20.3%)	Not informed	n=371 (10.2%)	Aggression, n=1,767 (48.6%)
Colli et al., 1997^16^	Ribeirão Preto, São Paulo, Brazil	Cross-sectional	General	Mild, n=2,584 (74.5%)	2476	71.4%	992	28.6%	0-10 years old (about 30% of all men about 10% of all women)	Not informed	n=209 (6%)	Traffic accidents, n=1,241(35.8%)
Gennari et al., 1995^17^	São Paulo, Brazil	Prospective	General	Mild, n=47 (47%)	85	85%	15	15%	Closed head injury patients’ mean age: 35.4 years old Penetrating head injury patients’ mean age: 27.2 years old	Not informed	n=12 (12%)	Traffic accidents, n=40 (40%)
Masini et al., 1994^30^	Distrito Federal, Brazil	Retrospective	General	Mild, n=76 (76%) (Independent 100 people sample)	65 (Independent 100 people sample)	65% (Independent 100 people sample)	35 (Independent 100 people sample)	35% (Independent 100 people sample)	1-30 years old, n=72 (72%) (Independent 100 people sample)	Not informed	n=797 (14.7%)	Traffic accident, n=2391 (44%)
Maset et al., 1993^18^	Sao Jose do Rio Preto, São Paulo, Brazil	Cross-sectional	General	Not informed	759	70.0%	325	30.0%	20-29 years old, n=303 (28.0%)	Not informed	Full text was not retrievable	Full text was not retrievable

We also extracted information on the consequences of TBI, patients’ clinical comorbidities, length of hospital stay, and alcohol consumption (Table 5). Surprisingly, many studies did not collect any neuroimaging findings, probably because neuroimaging is often not performed in mild TBI cases.^11^ ^,^ ^17^ ^,^ ^18^ ^,^ ^23^ ^,^ ^25^ ^,^ ^28^ ^,^ ^29^ ^,^ ^31^ ^,^ ^34^ ^,^ ^38^. In relation to other clinical findings, TBI was often accompanied by other soft tissue lesions and limb fractures^16^ ^,^ ^21^ ^,^ ^22^ ^,^ ^29^ ^,^ ^35^ ^,^ ^36^ ^,^ ^37^. Alcohol consumption ranged from 11.7 to 42.3%^15^ ^,^ ^16^ ^,^ ^23^ ^,^ ^24^ ^,^ ^29^ ^,^ ^36^.

**Table 5.** Traumatic brain injury-related consequences, clinical comorbidities, length of hospital stay, and alcohol intake information in epidemiological studies on traumatic brain injury.

Reference	Neuroimaging findings	Other clinical comorbidities/findings	Hospital stay length	Alcohol intake
Melo et al., 2019^24^	Computerized tomography, n=83 (70.9%) reported no encephalic lesions. From the remaining patients: (i), n=18 (15.4%) presented frontal lobe lesions; (ii), n=12 (10.3%) presented parietal lobe lesions; (iii), n=7 (6%) presented temporal lobe lesions; (iv), n=4 (3.4%) presented occipital lobe lesions.	Not informed	Not informed	19.7% (n=23) of patients displayed intoxication signs, according to their records. The remaining records did not include any information on patients’ alcoholic statuses.
Marinho et al., 2017^31^	Not informed	Not informed	Not informed	Not informed
De Almeida et al., 2016^11^	Not informed	Not informed	Mean hospital stay length for: (i) 2008: 5.4 days; (ii) 2009: 5.3 days; (iii) 2010: 5.5 days; (iv) 2011: 5.6 days; (v) 2012: 5.8 days. Overall mean length of hospital stays: 5.5 days.	Not informed
Vieira et al., 2016^15^	Early diffuse axonal injury and intracranial hypertension signs in computerized tomography are associated with greater mortality	Hypotension, hypertension, hypothermia, hyperthermia, hypoglycemia, hyperglicemia, bradycardia, tachycardia, and hypoxia.	Not informed	n=33 (42.3%) patients reported alcohol intake prior the trauma event.
Tavares et al., 2014^32^	Chronic subdural hematoma, n=63 (32.5%) Acute extradural hematoma, n=49 (25.3%) Acute subdural hematoma, n=30(15.5%) Cerebral edema, n=2 (1.0%) Firearm projectile, n=7 (3.6%) Depressed skull fracture, n=38 (19.6%) Intraparenchymal hematoma, n=5 (2.6%)	Not informed	Not informed	Not informed
De Souza et al., 2013^19^	Study conducted on TBI caused by projectile firearms Frontal lobe lesion, n=49 (27%) Temporal lobe lesion, n=45 (25%) Parietal lobe lesion, n=25 (14%) Occipital lobe lesion, n=31 (17%) Facial lesion, n=20 (11%) Multiple lesions, n=11 (6%)	Tangential TBI, n=29 (16%) Penetrating TBI, n=152 (84%)	Not informed	Not informed
Santos et al., 2013^25^	Not informed	Not informed	Not informed	Not informed
Fernandes et al., 2013^33^	Study did not specify whether lesions were chronic or acute. Fractures, n=11,125 (2.5%) Extradural hematoma, n=20,923 (4.8%) Subdural hematoma, n=27,447 (6.3%) Focal lesions, n=31,644 (7.2%) Diffuse lesions, n=159,241 (36.3%) Subarachnoid hemorrhage, n=1,856 (0.4%) Non-specified lesions, n=186,742 (42.5%)	Not informed	Not informed	Not informed
Carvalho Viégas et al., 2013^34^	Not informed	Not informed	Not informed	Not informed
Ruy and Rosa, 2011^35^	Not informed	Sensory reduction, n=45 (48.5%) Anisocoria, n=15 (16.3%) Mental confusion, n=11 (12.1%) Psychomotor agitation, n=10 (10.9%) Cardiopulmonary arrest, n=10 (10.9%) Respiratory failure, n=9 (9.8%) Seizures, n=6 (6.7%) ICU clinical complications: Pneumonia, n=16 (17.3%) Sepsis, n=2 (2.2%) Acute renal failure, n=2 (2.2%) Cerebral hemorrhage, n=36 (38.9%) Cerebral contusion, n=36 (38.5%) Cerebral edema, n=23 (24.9%) Bone fracture of any kind, n=18 (19.6%) Pneumocephalus, n=12 (12.9%)	Not informed	Not informed
Moura et al., 2011^26^	Study did not specify whether lesions were acute or chronic. Diffuse axonal injury, n=1 (0.99%) Extradural hematoma, n=20 (19.82%) Cerebral contusion, n=18 (17.82%) Subarachnoid hemorrhage, n=10 (9.9%) Subdural hematoma, n=6 (5.94%) Most afflicted cranial sites: Frontal, n=25 (24.75%) Temporal, n=12 (11.88%) Temporoparietal, n=12 (11.88%) Parietal, n=9 (8.91%) Occipital, n=6 (5.94%) Parietofrontal, n=6 (5.94%) Frontotemporal, n=4 (3.96%) Temporooccipital, n=2 (1.98%) Basilar skull fracture, n=2 (1.98%)	At admission: Headache, n=17 (16.83%) Vomiting, n=16 (15.84%) Otorrhagia, n=9 (8.91%) Coma, n=6 (5.94%)	Not informed	Not informed
Ramos et al., 2010^36^	General nervous system lesion, n=34 (19.9%)	Bone lesion, n=39 (22.8%) Vascular lesion, n=55 (32.2%) Multiple lesions, n=26 (15.2%) Soft tissues, n=7 (4.1%)	Not informed	n=20 (11.7%)
Guerra et al., 2010^21^ *(Only severe TBI) cases were analyzed)*	Diffuse Axonal Injury, n=56 (42.4%) Swelling, n=74 (56.1%) Intraparenchymal hemorrhage, n=46 (34.8%) Subarachnoid hemorrhage, n=41 (31.1%) Study did not specify whether lesions were acute or chronic: Subdural hematoma, n=20 (15.2%) Intraventricular hemorrhage, n=15 (11.4%) Extradural hematoma, n=14 (10.6%)	Thoracic lesion, n=48 (36.4%) Skeletal muscle lesion, n=37 (28.0%) Abdomen, n=21 (15.9%) Spinal cord, n=6 (4.6%)	Not informed	Not informed
Martins et al., 2009^37^ *(Only severe TBI cases were analyzed)*	Marshall type I injury, n=22 (2.9%) Marshall type II injury, n=175 (23.4%) Marshall type III injury, n=172 (23.0%) Marshall type IV injury, n=58 (7.8%) Evacuated mass lesion, n=240 (32.1%) Non-evacuated lesion, n=30 (4.0%) Brainstem lesion, n=50 (6.7%) Subarachnoid hemorrhage, n=267 (35.7%)	Face trauma, n=108 (14.4%) Cervical spine trauma, n=27 (3.6%) Dorsal-lumbar spine trauma, n=7 (0.9%) Thoracic trauma, n=141 (18.9%) Abdominal trauma, n=70 (9.4%) Limb trauma, n=204 (27.3%) (Pupil) Isochoric, n=283 (37.8%) (Pupil) Miotics, n=30 (4.0%) (Pupil) Anisocorics, n=347 (46.4%) (Pupil) Mydriatics, n=83 (11.1%)	Not informed	Not informed
Braga et al., 2008^23^ *(Only TBI cases caused by falling from standing height were analyzed)*	Not informed	Systemic arterial hypertension, n=9 (11.8%) Epilepsy, n=6 (7.9%) Alcoholism, n=4 (5.3%) Diabetes mellitus, n=3 (3.9) Heart failure, n=3 (3.9%) Alzheimer’s disease, n=3 (3.9%) HIV infection, n=3 (3.9%)	Not informed	n=11 (14.5%)
Faria et al., 2008^38^	Not informed	Not informed	Not informed	n=33 (39.3%)
Pereira et al., 2006^27^	Altered CT scan, n=75 (31.0%) out of 242 Altered plain radiography of the skull, n=4 (1.7%) out of 239	Altered conscious level, n=85 (18.1%) Vomiting and nausea, n=97 (20.6%) Sleepiness, n=51 (10.9%) Headache, n=40 (8.5%) Dizziness, n=18 (3.8%) Seizures, n=11 (2.3%) Otorrhagia, n=12 (2.6%) Epistaxis, n=8 (1.7%) Diplopia, n=2 (0.43%)	Not informed	Not informed
Melo et al., 2006^28^ *(Study conducted on children and teenagers only)*	Not informed	Not informed	Not informed	Not applicable
Melo et al., 2004^29^	Not informed	1 lesioned organ, n=117 (66.1%) 2 lesioned organs, n=40 (22.6%) ≥3 lesioned organs, n=20 (11.3%)	Not informed	n=27 (4.9%)
Dantas Filho et al., 2004^39^	Marshall type I injury, n=15 (7.28%) Marshall type II injury, n=63 (30.58%) Marshall type III injury, n=33 (16.02%) Marshall type IV injury, n=13 (6.31%) Focal lesion (operated), n=72 (34.95%) Focal lesion (not operated), n=10 (4.85%)	Hypo-/Hypernatremia and Hypo-/hypercalcemia, n=130 (63.21%) Polyuria, n=32 (15.53%) Bronchopneumonia, n=119 (57.77%) Urinary infection, n=11 (5.34%) Sepsis, n=10 (4.85%) Sinusitis, n=6 (2.91%) Gastrointestinal bleeding, n=3 (1.46%) Hypoxia, n=81 (39.32%) Hypotension, n=39 (18.93%) Both hypoxia and hypotension, n=22 (10.68%)	Not informed	Not informed
Gusmão et al., 2002^22^ (*Only evaluated fatal patients)*	Diffuse axonal injury, n=96 (80.0%) Intracranial hypertension, n=47 (39.2%) Skull fracture, n=63 (52.5%) Hypoxic brain injury: (19.2%)	Limb fractures, n=46 (38.3%) Thoracic trauma, n=42 (35%) Abdominal trauma, n=44 (36.7%) Both thoracic and abdominal trauma, n=32 (26.7%) Pneumonia, n=10 (8.3%) Purulent meningitis, n=3 (2.5%)	Not informed	Not informed
Koizumi et al., 2001^40^ *(Only evaluated children)*	Skull fractures, n=1,800 (11%)	Not informed	<1 day, n=333 (2.0%) 1 to 3 days, n=12,100 (73.9%) 4 to 7 days, n=2.825 (17.3%) 8 to 29 days, n=1.023 (6.2%) ≥30 days, n=95 (0.6%)	Not applicable
Koizumi et al., 2000^20^	Fracture of skull vault, n=45 (1.2%) Basilar skull fracture, n=32 (0.9%) Other skull fractures, n=22 (0.6%) Multiple fractures of skull/face, n=4 (0.1) Brain concussion, n=1038 (28.6%) Cerebral laceration and contusion, n=192 (5.3%) Hemorrhage, n=509 (14.0%) Traumatic intracranial lesion of other types, n=1793 (49.3%)	Not informed	Most predominant hospital stay duration is of 1 to 7 days hospitalized (n=2,637; 72.5%).	Not informed
Colli et al., 1997^16^	Plain radiography of the skull: 18.0% (24% of 73%) presented fractures. CT scan: 4.2% (30% of 14%) presented brain lesions	Scalp lesion: 66.2% Headache (21.4% of children) Vomit: 17% (approximately in adult and children) Headache: 17% (approximately) Alteration of consciousness (some time after TBI): 24.4% Alteration of consciousness (immediately afterwards): 87% Soft tissue lesion: 17.9% Face lesion: 15.4%* *Full text was not retrievable. Figure 7 was missing	Not informed	17% of adults (approximately)*
Gennari et al., 1995^17^	Not informed	Penetrating trauma, n=32 (32%) Blunt trauma, n=68 (68%)	Not informed	Not informed
Masini et al., 1994^30^	Chronic subdural hematoma, n=54 (1) Acute extradural hematoma, n=40 (0.7%) Acute subdural hematoma, n=40 (0.7%) General fractures and basilar skull fracture, n=58 (1%) Cerebral contusion, n=56 (1%) Firearm projectile induced lesion: 19 (0.4%) Intracerebral hematoma: 9 (0.2%)	Not informed	n=64 (64%) were discharged <24 hours. n=16 (16%) stayed longer than 7 days.* *Independent 100 people sample 71.6% patients	Not informed
Maset et al., 1993^18^	Not informed	Not informed	Average hospital stays: 4.65 days 71.6% patients stayed for a maximum of 4 days. 24.9% patients stayed for 2 days. 1.7% patients stayed for a period greater than 20 days.	Not informed

TBI: traumatic brain injury

## DISCUSSION

In the present study, we evaluated the sociodemographic and clinical characteristics of patients with TBI admitted to a public reference trauma center in Minas Gerais, Brazil. This is the first study to perform such evaluation in the state of Minas Gerais, specifically at one of the largest reference trauma centers in Brazil. It is worth highlighting the large number of patients admitted to this center in a short period of time. The hospital admitted almost 17 patients with TBI every day. Young men were most commonly affected, and unspecific falls were the most common cause of TBI. Overall, these findings are consistent with the results of other Brazilian studies, as shown in our systematic literature review ^11^ ^,^ ^15^ ^-^ ^40^.

The higher vulnerability of men can be explained by sociocultural and behavioral factors, such as higher exposure to urban violence than women^25^. A European systematic review found a preponderance of men in 28 studies in which the male-to-female ratio ranged from 1.2:1.0 to 4.6:1.0^41^. Accordingly, men in the United States have higher age-adjusted rates of emergency department visits and deaths related to TBI^4^. In our sample, TBI occurred more frequently in young adults, with mean ages ranging from 22 to 49 years in different studies^25^ ^,^ ^41^ ^,^ ^42^.

In contrast to most Brazilian reports, the current study found that falls were the main cause of TBI, but not traffic accidents^29^ ^,^ ^43^. One of the largest epidemiological studies conducted in the Brazilian population found that falls were the most common TBI mechanism, similar to our findings^33^. Falls were also the most common cause of TBI in European countries and in the USA^4^ ^,^ ^41^.

Approximately 19% of our sample reported having consumed alcohol prior to the traumatic event. Our results show that falls, followed by traffic accidents, were the main causes of TBI in patients under the influence of alcohol. Falls were also the main cause of TBI in patients under the influence of illicit drugs (mainly marijuana and crack), but here traffic accident was followed by physical aggression. It is known that the use of alcohol and illicit drugs favors the occurrence of risky situations^29^. In an American study, it was found that both alcohol and illicit drug use were common before a TBI^43^. In Brazil, it is still unclear what role alcohol and other drugs play in TBI^36^. Most of the studies included in our review did not evaluate alcohol status of patients, and those that did had missing data on such information^15^ ^,^ ^16^ ^,^ ^23^ ^,^ ^29^ ^,^ ^36^.

Regarding the severity of TBI, as determined by the GCS, the majority of our sample was diagnosed as mild (87.4%). Mild TBI was also the most common severity level in the Brazilian studies examined, but studies differed in their sample composition. For example, Marinho et al. analyzed a group of 18-30-year-old individuals - an age group more prone to riskier situations and to moderate and severe TBI^29^ ^,^ ^31^ ^,^ ^43^. Faria et al. grouped severe and moderate TBI together and yet accounted for only 52% of the total cases^38^.

The clinical meaning of mild TBI should not be underestimated, as it has been associated with the development of cognitive and behavioral changes^44^. According to one scoping review, half of patients with a single episode of mild TBI develop long-term impairments in several cognitive domains, including executive functions, learning/memory, attention, processing speed, and language^45^. This review included heterogeneous studies using different cognitive batteries in mild TBI patients at different time points after the traumatic event, which may explain the high rate of cognitive deficits. For example, significant episodic memory deficits can already be observed in the acute phase of mild TBI^46^.

Neuroimaging is an important tool in establishing the prognosis for TBI. Seventy-six of 436 (17.4%) patients had early tomographic/neuroimaging TBI-related alterations. It is well known that the more severe the TBI, the more likely the patient is to have neuroimaging changes^47^. Our results confirm that more than half of the patients with moderate or severe TBI had cranial CT changes. Conversely, about 10% of patients with mild TBI had neuroimaging changes. Few of the Brazilian studies reviewed included their neuroimaging findings, as neuroimaging is not considered cost-effective due to the low rate of positive neuroimaging findings in mild TBI^48^.

The length of hospital stay was less than 24 hours in 73.6% of the cases, as most were mild TBI cases. Conversely, a GCS score of 12 or less on admission, as well as neuroimaging changes and medical comorbidity (i.e., both clinical and psychiatric conditions), were associated with a longer hospital stay. Similar to our results, Sorensen et al. found that lower GCS score and psychiatric comorbidity were significantly associated with delay in hospital discharge in patients with TBI^49^. The length of hospital stay in our systematic review varied widely, probably due to the heterogeneity of the sample and the different protocols for treatment and management of TBI in different clinical settings.

In the current study, 3.6% of our post-TBI patients died (n=18). Mortality rates should be interpreted with caution, considering the heterogeneity of epidemiological studies on TBI. For instance, Fernandes et al. found a mortality rate of 12.0% in a much larger sample that included over 400,000 records from a much longer time window^33^. In Europe, there is also a wide variation in post-trauma mortality rate, ranging from 3.0/10^5^ inhabitants per year in both Hannover and Münster (Germany) to 18.3/10^5^ per year in Finland and Italy^41^. In the USA, about one third of all related deaths are diagnosed with TBI^50^.

There are limitations to the present study. Some variables (e.g., level of education) were not available for a significant percentage of patients, reflecting the challenges of clinical data collection in a busy trauma center, and thus preventing a more thorough analysis. Medical records also did not include categories of falls. We were only able to capture serious sequelae during hospitalization, which prevented us from exploring less severe complications, including cognitive, behavioral or motor symptoms, and the associated impact on patients’ lives. In addition, the present study was conducted in a time window of one month within one year - which was one of the main reasons that led us to conduct a systematic review. From the literature review, we obtained an accurate snapshot of TBI epidemiology in one of the main trauma centers in one of the largest metropolitan regions of Brazil. We chose the month of July because of winter break - a time of year in which people are more exposed to risky situations (such as car travel) and, consequently, to TBIs.

Future studies with a comprehensive longitudinal evaluation of TBI beyond the acute phase are warranted. The investigation of regional specificities in TBI profile in other Brazilian regions and other developing countries could also provide meaningful clinical and epidemiological information. Only with robust evidence can optimal prevention and rehabilitation measures be implemented, influencing the outcome of this daunting problem.
